# Structure and activity alteration in adult highland residents' cerebrum: Voxel-based morphometry and amplitude of low-frequency fluctuation study

**DOI:** 10.3389/fnins.2022.1035308

**Published:** 2022-11-24

**Authors:** Minzhi Zhong, Huaqu Zeng, Dongye Wang, Jiesheng Li, Xuguang Duan, Yong Li

**Affiliations:** ^1^Department of Radiology, Guangzhou Red Cross Hospital, Guangzhou, China; ^2^Department of Radiotherapy Center, Gaozhou People's Hospital, Guangdong, China; ^3^Department of Radiology, Sun Yat-sen Memorial Hospital, Guangzhou, China; ^4^Department of Radiology, Sanshui People's Hospital, Foshan, China; ^5^Department of Radiology, Nyingchi People's Hospital of Tibet Autonomous Region, Nyingchi, China

**Keywords:** high altitude, structure, function, cerebrum, fMRI

## Abstract

**Introduction:**

People living in highland areas may have factors that allow them to adapt to chronic hypoxia, but these physiological mechanisms remain unclear. This study aimed to investigate the brain mechanism in a cohort of adult residents of Tibet, a well-known plateau section in China, by observing differences in brain structure and function in non-plateau populations.

**Methods:**

The study included 27 Tibetan and 27 non-plateau region residents who were matched in age, sex, and education. All participants underwent high-resolution three-dimensional T1 weighted imaging (3D-T1WI) and resting-state functional magnetic resonance imaging (rs-fMRI) scans on a 1.5 Tesla MR. Gray matter volumes and regional spontaneous neuronal activity (SNA) were calculated and compared between the two groups.

**Results:**

When comparing gray matter in people living in high altitudes to those living in the flatlands, the results showed positive activation of gray matter in local brain regions (*p* < 0.05, false discovery rate (FDR) corrected), in the right postcentral [automated atomic labeling (aal)], left postcentral (aal), and right lingual (aal) regions. Comparing the people of high altitude vs. flat land in the brain function study (*p* < 0.05, FDR corrected), positive activation was found in the right superior motor area (aal) and left superior frontal (aal), and negative activation was found in the right precuneus (aal).

**Conclusion:**

In high-altitude individuals, larger regional gray matter volumes and higher SNA may represent a compensatory mechanism to adapt to chronic hypoxia.

## Introduction

Hypoxia is a common phenomenon and is characterized by a decrease in oxygen content in cells or tissues relative to normal levels (Zhang et al., 2022). The brain is highly sensitive to changes in oxygen content because the central nervous system is highly oxidized (Otero-Losada et al., 2019; Burtscher et al., 2021; Li et al., 2021). The effects of hypoxia on the nervous system are related to the severity of systemic hypoxia and the differential expression patterns of neurogenesis factors at maturity (Johnston, 1995; Schneider et al., 2012; Chen and Gaber, 2021).

Acute hypoxia usually presents obvious neurological symptoms, which are of concern to us, whereas the effects of chronic hypoxia on the body do not appear immediately and are often overlooked. Some studies have pointed out that elderly people living for a long time in a low-oxygen environment have a higher incidence of Alzheimer's disease than in a plain area, which suggests that environmental factors have an important impact on the onset of cognitive impairment (Ma et al., 2014). The hypoxia symptoms, such as anxiety, depression, and cognitive impairment, have been reported mainly in the migrant population, but less so in the locals living on the plateau (Tibetans). Mammals have evolved physiological mechanisms to cope with hypoxia, including increased ventilation, cardiac output, vascular growth, and the number of circulating red blood cells (McClelland and Scott, 2019; Lee et al., 2020; Biller et al., 2021). Compared with people living in plain areas, Tibetans exposed to changes in the acute oxygen environment have an obvious cerebral hemodynamic regulation mode (Wu and Kayser, 2006; Xing et al., 2019). Hypoxia activates a variety of epigenetic mechanisms in the fetal brain, increasing the vulnerability of the offspring to neurodevelopmental disorders (Li et al., 2006; Butt et al., 2021). Acute and chronic hypoxia elicits many responses at the cellular level, with an overall decline in oxygen consumption with age. The adjustment of the adaptive mechanisms that arise in the brain is unclear. The anti-oxidative stress theory proposed by scholars to protect the cognitive function of people at high altitudes remains uncertain, and the extent to which chronic hypoxia affects the body is unclear.

Voxel-based morphometry (VBM) is a hypothesis-free, whole-brain, voxel-by-voxel analytical method that attempts to compare imaging data between populations (Melonakos et al., 2011). It is a technology used to analyze brain structures (Richardson et al., 2011; Herrera and González-Candia, 2021). It was used to generate 3D high-resolution T1 images that were used for segmentation to obtain gray matter, white matter, and cerebrospinal fluid images to analyze brain microstructure (Bashir et al., 2019). The amplitude of low-frequency fluctuation (ALFF) is used for resting-state functional magnetic resonance imaging (fMRI) performed by the Data Processing and Analysis of Brain Imaging (DPABI) software to detect blood flow changes associated with neural activity in different tasks by detecting local blood-oxygen level signals (Vanasse et al., 2018).

By gaining an in-depth understanding of the mechanisms of various hypoxia patterns, we hoped to reduce the impact on the human body and find the beneficial aspects of hypoxia in humans. In our study, 54 healthy adults were enrolled and underwent magnetic resonance imaging (MRI) for structural and functional studies. The aim of this study was to investigate the effects of chronic hypoxia on the brains of healthy individuals to understand the mechanism of the brain's response to hypoxia.

## Materials and methods

### Participants and procedures

There were 27 participants (11 men) in the high-altitude group (HA) enrolled in the study, aged 35–45 years (mean: 38.65 ± 5.4). The average schooling year of HA was 12.04 ± 0.6 years. The control group (CG) consisted of 27 (13 men) medical staff who had been in Tibet for <2 weeks, aged 28–45 years (mean: 41.77 ± 6.4) years, with an average schooling year of 12.52 ± 3.0 years. All participants were informed of the purpose and procedures of the study, and informed consent was obtained from all participants.

The HA had lived at an altitude of over 2,800 m for more than 30 years. The CG had lived in the flatlands of Tibet for less than 2 weeks. The inclusion criteria were as follows: (1) right hand; (2) no respiratory symptoms and cardiopulmonary diseases, history of diabetes, and hypertension; (3) no abnormal brain structure found on routine series MRI; (4) no history of neurological disease or cognitive decline; (5) Mini-Mental State Examination (MMSE) score >28; and (6) test for oxyhemoglobin saturation (SpO_2_) and hemoglobin (Hb).

### MRI data equipment

All subjects underwent brain MRI scans using a GE Medical Systems 1.5T-scanner (GE, Signa, USA) at the Department of Diagnostic Imaging in Linzhi People's Hospital of Tibet with an 8-channel head coil in 2018. When routine protocols used to scan the brain had no organ disease, high-resolution T1-weighted images (BRAVO) with good contrast between gray and white matter were collected. The following image parameters were included: TR = 12.5 ms, TE = 5.2 ms, flip angle = 5^o^, slice thickness =1 mm, TI = 1100 ms, 130 slices, matrix =1 × 1 × 1 mm^3^, field of view =240 × 240 mm. The gradient echo-planar imaging (EPI) sequence was blood oxygen level-dependent (BOLD) for resting-state functional MRI as follows: TR = 3,000 ms, TE = 40 ms, flip angle = 90^o^, 33 slices, slice thickness = 3 mm, resolution =64 × 46 × 5 mm^3^, time-point 128, the field of view= 240 × 240 mm.

### Image processing

All data processing was performed on the Matlab version R2013a platform (nl.mathworks.com/products/matlab/) (Yan et al., 2016; Chen et al., 2022).

Brain volume (BRAVO) imaging was subjected to voxel-based morphometry (VBM) analysis and was performed using Statistical Parametric Mapping (SPM8, The Wellcome Center for Human Neuroimaging, London, UK; http://www.fil.ion.ucl.ac.uk/spm) running under MATLAB software (The Mathworks, Inc., Natick, MA, USA). All 3D-T1 images were corrected for rough bias, affine registered to a template image in the Montreal Neurology Institute (MNI) space, and then segmented into white matter (WM), gray matter (GM), and cerebrospinal fluid (CSF) maps. The segmented images were spatially normalized by high-dimensional diffeomorphic anatomical registration using the DARTEL algorithm. Finally, all normalized images of regional gray matter volume were smoothed with an 8-mm full-width at half maximum (FWHM) Gaussian kernel to improve the signal-to-noise ratio.

BOLD imaging was performed using DPABI (http://rfmri.org/dpabi). First, the first 10 s were removed to allow the magnetization to reach a steady state. The following main steps were included: (1) slice timing to head motion correction; (2) realignment; (3) normalization to the MNI coordinate space with 3 Ã−3 Ã−3 mm^3^; (4) linear detrending; (5) band-pass filtering (0.01–0.08 Hz); and (6) nuisance signals, which regressed out the signal including white matter and cerebrospinal fluid. Subsequently, spatial smoothing with a 6-mm FWHM isotropic Gaussian kernel was performed to reduce the noise.

### Neuropsychiatric test

The Mini-Mental State Examination (MMSE) score includes orientation, memory, attention, calculation, recall ability, and language ability. The total possible score was 30 points. A score of <27 points indicated cognitive dysfunction.

### Statistical analysis

All demographic and clinical data were analyzed using IBM SPSS Statistics 25. A two-sample *t*-test was used to compare age, education, pulse oximetry [SpO_2_ (%)], MMSE score, and hemoglobin level. To determine the differences between HA and CG, two-sample *t*-tests were performed on gray matter and function images: voxel-level, *p* < 0.05; cluster size >200; voxel number = 5,000; and false discovery rate (FDR) correction. Spearman's correlation analysis was conducted to observe the relationship between the clinical hemoglobin level and activation in the VBM and ALFF values. The statistical significance level was set at *p* < 0.05.

## Results

### Demographic and clinical characteristics

**Figure 1:**
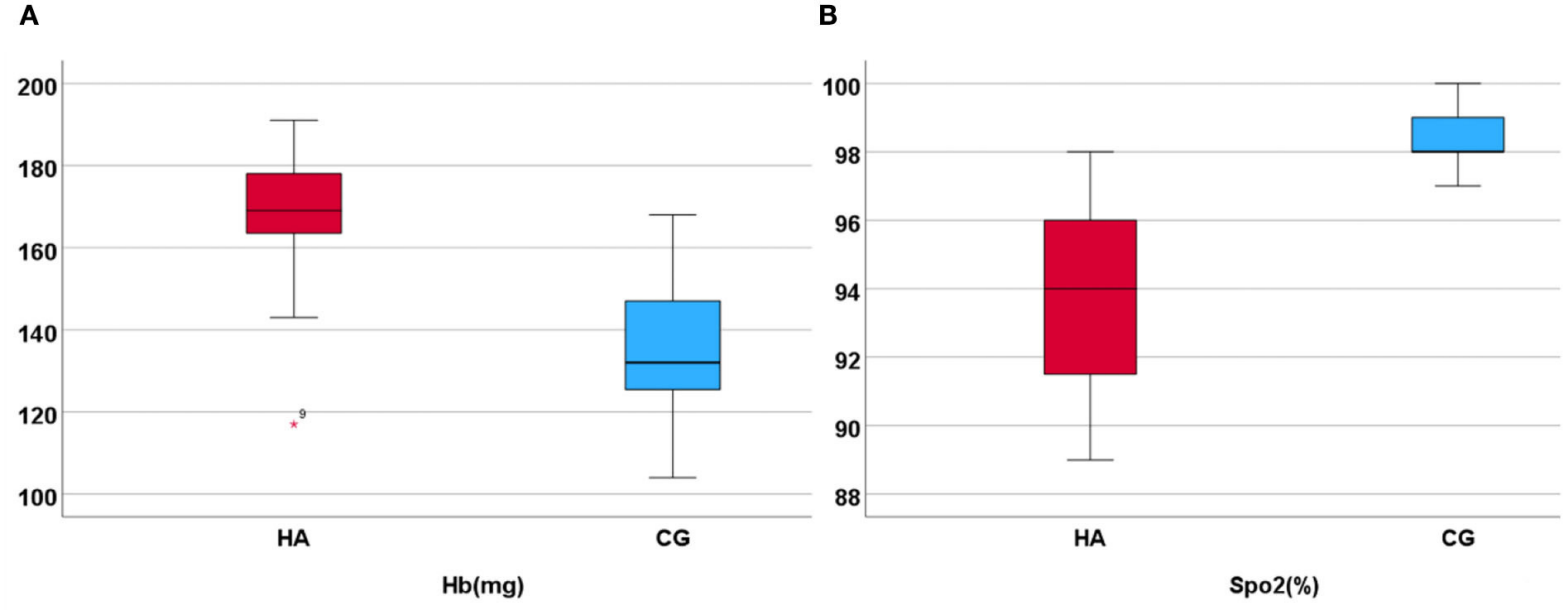
Hb and SpO_2_ in HA and CG show that the Hb is higher in HA than in CG, but the SpO_2_ (%) is lower. **(A)** Display the hemoglobin higher in high altitude group. **(B)** Display the SpO_2_ higher in control group.

The clinical and demographic characteristics of the participants are presented in Table 1, which are included in the analysis. The MMSE test scores of all participants were >29. Of the total participants, 25 men (92.6%) graduated from high school or above. There was no significant difference in age, education, and MMSE scores between the HA and CG. The SpO_2_ value was 93 ± 2.5% and the Hb value was 169 ± 14.6 mg in HA; the SpO_2_ value was 98.0 ± 0.92% and the Hb value was 135 ± 16.8 mg in CG (Figure 1). The values of Hb and SpO_2_ (%) were significantly different between the HA group and CG (*p* < 0.05) (Table 1).

**Table 1:** Demographic, clinical data of the HA and CG.

	**HA**	**CG**	***p*-value**
Age (years)	38.65 ± 5.4	41.77 ± 6.4	0.079
Gender (male, female)	11, 40.7%, 16, 59.2%	13, 48.1%, 14, 51.9%	–
Education (years)	12.04 ± 0.6	12.52 ± 3.0	0.554
Hb (mg)	169 ± 14.6	135 ± 16.8	<0.000
SpO_2_ (%)	93 ± 2.5	98.0 ± 0.92	<0.000
MMSE score	29.22 ± 0.6	29.65 ± 0.6	0.067

All data were recorded as mean ± standard deviation (SD).

### VBM between HA and CG

**Figure 2:**
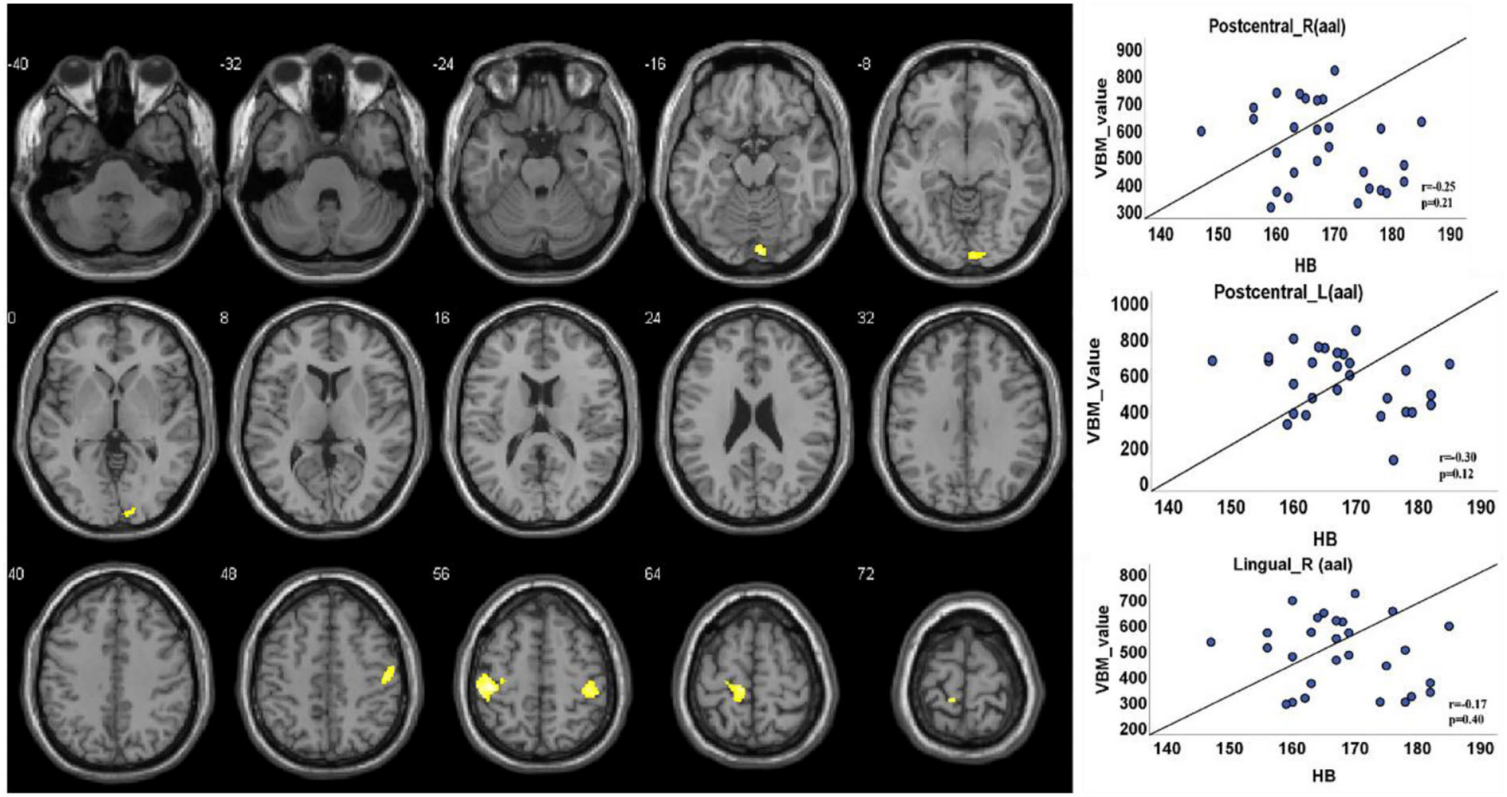
Gray matter structure cluster area differs from HA to CG and uncorrelates with Hb. The volume is significantly higher with the people of HA than CG. The color yellow represents the brain regions that positively activate gray matter. Correlations with Hb: Right lingual (*r* = 0.17, *p* = 0.40); right postcentral (*r* = 0.25, *p* = 0.21); left postcentral (*r* = 0.30, *p* = 0.12).

The VBM analysis revealed a significantly increased volume of gray matter in HA compared to CG in the bilateral somatosensory cortex (Brodmann 3, left, t = 4.88; right, *t* = 4.26, FDR corrected, cluster size number >200, *p* < 0.05) and the vision cortex (Brodmann 18, *t* = 4.26, FDR corrected, cluster size number >200, *p* < 0.05; Figure 2, Table 2). This showed a uncorrelation between changes in gray structure and Hb.

**Table 2:** VBM analysis of significant GM cluster in HA vs. CG.

**Brain region**	**BA**	**Cluster size (mm^3^)**	***t***	**MNI**		
				***X***	***Y***	***Z***
Left postcentral (aal)	3	466	4.88	−45.5	−34	57
Right postcentral (aal)	3	409	4.26	39	−28	57
Right lingual (aal)	18	403	4.32	21.6	−90.3	−13.5

t: peak value; MNI: coordinates established for MRI images of the normal human brain. The coordinates x, y, and z refer to the anatomical location.

### ALFF between HA and CG

**Figure 3:**
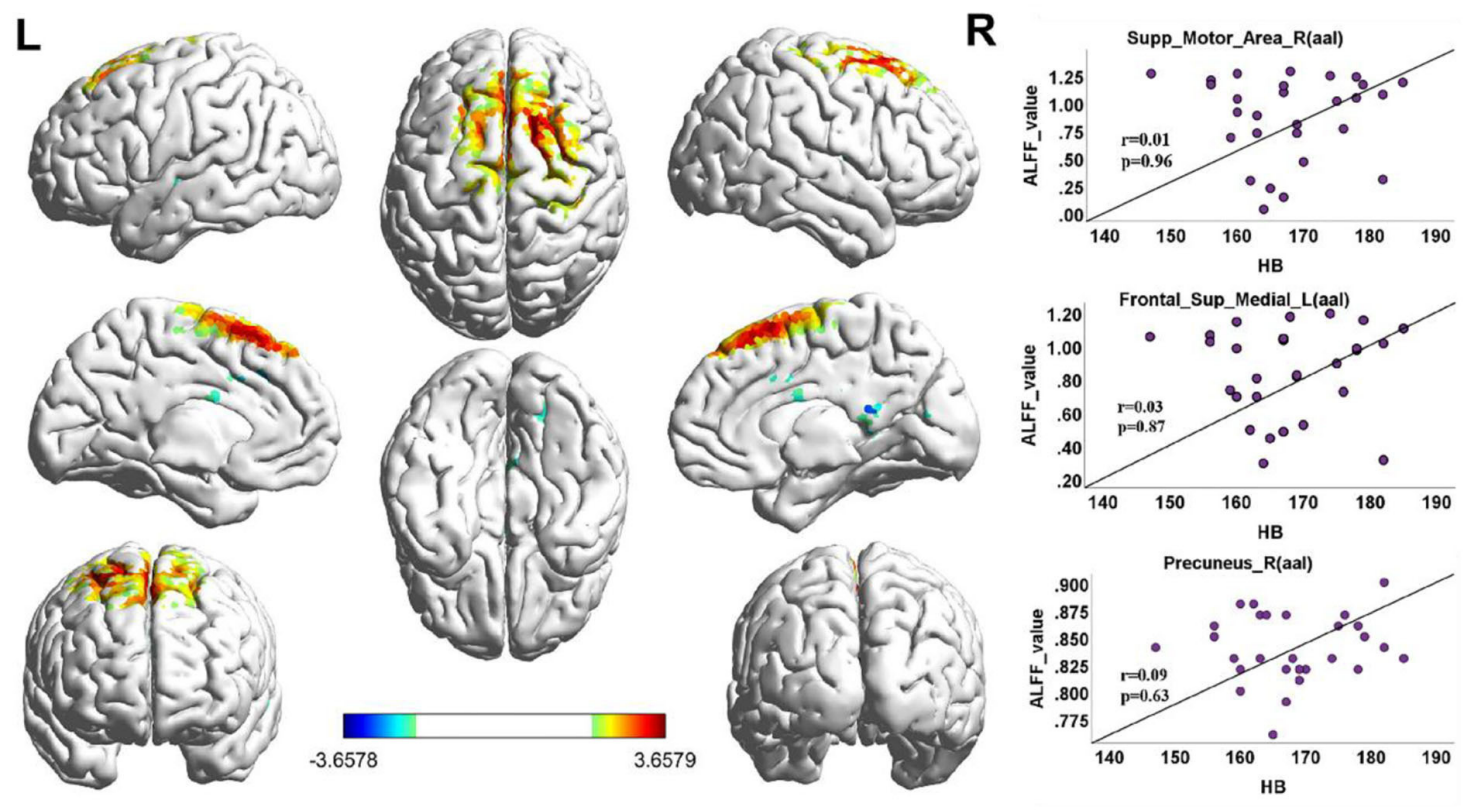
ALFF cluster area and the correlation with Hb, dark red with a significantly increased value, and dark blue with a significantly decreased value; the color bar represents the *t*-score. ALFF value correlation with Hb in Spearman. Correlation diagrams display the right superior motor area (aal; *r* = 0.01, *p* = 0.96). Left frontal superior (aal; *r* = 0.03, *p* = 0.87); right precuneus (aal; *r* = 0.09, *p* = 0.63).

When the functional area with HA was compared to CG, there was positive activity in the Brodmann 6 and Brodmann 8 regions of the brain in the upper motor cortex, in the Brodmann 18 region (Figure 3, Table 3).

**Table 3:** Between-group differences in the function area.

**Brain region**	**BA**	**Cluster size**	***t***	**MNI**		
				***X***	***Y***	***Z***
Right superior motor area (aal)	6	140	3.50	16	14	57
Left superior frontal cortex (aal)	8	153	3.60	−9	55	52
Right precuneus (aal)	18	52	−4.08	12	−51	18

R, right; L, left; BA, Brodmann area; MNI, coordinates established for MRI images of the normal human brain; AlphaSim correction, cluster size number > 200; p_value_ > 0.01.

## Discussion

Tibetans are known to have high hemoglobin levels, a hallmark feature of adaptation to high-altitude exposure (Chen et al., 2022). Previous studies have suggested that Hb concentration is closely related to the severity of leukoaraiosis and is an independent factor with a positive correlation (Long and Xiuli, 2012; Tang et al., 2012). However, plateau and plain residents have completely different adaptations to hypoxia (Yonglan, 2021). In this study, we found that gray matter volume decreased in parts of the cerebral cortex in HA, which were the bilateral postcentral cortex and the right lingual cortex. The results showed that compared with the CG group, the right superior motor area and left superior frontal cortex ALFF increased in the HA group, and the right precuneus decreased. These results uncorrelated with low Hb levels.

The lingual gyrus is the occipital visual cortex that processes visual information. It is at the core of visual analysis and plays an important role in visuospatial perception. Somatosensory systems have commonalities with auditory and visual cortical functional units and conduction pathways, and mutual compensatory functions exist among the three (Zhang et al., 2018). Each retinal point responds to a portion of its visual cortex; hypoxia is an important factor and is related to the longer time that hypoxia factors act on the body (Guoen and Rili, 2021; Haoyu and Qing, 2021; Zhang, 2021). In our results, both gray matter and ALFF were activated in the right lingual gyrus of the HA, suggesting that the structure and function have changed in the visual area of the brain. Yin et al. (2017) believed that ALFF decreased in the right lingual gyrus of normal adults 2 years after the migration to high altitudes, compared with that before migration. Experts point out that long-term living in high-altitude environments can cause retinal diseases due to chronic hypoxia, high ultraviolet radiation, strong wind and sand stimulation, and vitamin deficiency, such as macular degeneration and optic nerve damage that impair visual function (El Chehab et al., 2012; Runjia et al., 2013; Jing et al., 2019). This can explain the gray matter and functional changes in the occipital lingual gyrus in the high-altitude population in our study.

Postcentral is the first motion sensory area, which mainly collects sensory input from the limbs, reflecting that the sensitivity of that population was affected. It is related to tactile (pain, temperature, touch, pressure, and position) information of the entire body. The functional region of the occipital lobe is closely associated with the postcentral region; it is involved in attention and visuospatial perception, and there are a variety of nerve conduction pathways. In our study, there was a large decrease in gray matter in the lingual gyrus, consistent with previous studies (Li and Li, 2020). This helps us understand the mechanism of visual impairment in people at high altitudes. Meanwhile, the cluster of the postcentral gyrus was also obviously activated, perhaps because of various tactile changes in the body to adapt to the extreme high-altitude environment or occipital interaction with the regulation of postcentral gyrus function.

Functional studies have implicated the superior frontal cortex, including the supplementary motor area (SMA) and pre-supplementary motor area (preSMA), in movement and cognitive control (Caixia et al., 2014). Brodmann 8 is involved in controlling saccadic movement (Zhang et al., 2012). It plays an important role in receiving visual information from space and organizing eye motor commands toward it, which is related to the function of the retina (Watanabe, 2017). In high-altitude migrants, bilateral visual cortex mirror functional connectivity was significantly increased, which was positively correlated with hemoglobin concentration (Lanzilotto et al., 2013). The superior motor area in Brodmann type 6 is the premotor cortex. The supplementary motor area is involved in the planning and execution of intentional movements. Our studies were conducted on Brodmann 6 using ALFF processing, suggesting that exercise capacity is affected in high-altitude people. Chen et al. (2016) found that hypoxia reduces exercise capacity, which is related to the quantity of Hb and the affinity of Hb-O_2_. Jay et al. (2022) suggested that the motility of people at high altitudes is inversely correlated with the concentration of Hb. The higher the Hb value, the lower the motility. Some studies suggest that people from high-altitude areas migrate to low-altitude areas, and their athletic ability is better than that of residents living in low-altitude areas, which is related to the genetic variation of Tibetans in high-altitude areas (Tatum et al., 2015). Although the correlation between motility and Hb values was not analyzed in this study, the changes in cortical function associated with exercise in structural and functional studies were consistent with those of previous studies. The neural connection between them needs to be further evaluated.

In recent years, the precuneus has received considerable attention in the study of neural functions. It is believed that this cortex plays a central role in a wide range of highly integrated tasks, including visuospatial imaging, episodic memory retrieval, and self-processing. Brodmann 18 is the visual cortex, which, when impaired, causes several visual disorders, including visual field defects, metamorphopsia, and different kinds of visual agnosia (Chuanming et al., 2006; Bianba et al., 2014; Kawachi, 2017). Functional analysis of our study revealed that the right precuneus was negatively activated in ALFF, suggesting that the high-altitude population has weakened visual functions in this aspect, which is consistent with the structural analysis results.

## Conclusion

This study showed consistent changes in the structure and activity of the sensorimotor regions. The eyes and visual control areas have the same time-frequency, showing that the high-altitude population differs from the plain population in terms of athletic ability and vision in the physiological state. These differences may be related to changes in brain function caused by living conditions and the environment. There were no obvious clinical manifestations of cognition in our study, which may be affected by the volunteers being young at a high living and education level.

The next study will evaluate the white matter fibers of this group of people to perform functional connectivity (FC) analysis to understand the coordination between white matter pathways and functional areas. A limitation of this study might be the small number of participants. Second, all cases were tested at high altitudes; although we performed a simple mental quantification test, we could not rule out that mild hypoxia may cause short-term asymptomatic damage to the brain of the control group.

## Data availability statement

The raw data supporting the conclusions of this article will be made available by the authors, without undue reservation.

## Ethics statement

Ethical review and approval was not required for the study on human participants in accordance with the local legislation and institutional requirements. The patients/participants provided their written informed consent to participate in this study.

## Author contributions

MZ completed the study design, data collection, and article writing. XD completed the data collection work, MR image quality analysis, and chart making. HZ completed the statistical analysis. DW and JL mainly completed MR image quality analysis. YL participated in the revision of the manuscript. All authors contributed to the article and approved the submitted version.

## Funding

This work was supported by Traditional Chinese Medicine Research Project of Guangdong Traditional Chinese Medicine Bureau No. 20232132 and Nyingchi Science and Technology Bureau No. 2018KJYZ016.

## Conflict of interest

The authors declare that the research was conducted in the absence of any commercial or financial relationships that could be construed as a potential conflict of interest.

## Publisher's note

All claims expressed in this article are solely those of the authors and do not necessarily represent those of their affiliated organizations, or those of the publisher, the editors and the reviewers. Any product that may be evaluated in this article, or claim that may be made by its manufacturer, is not guaranteed or endorsed by the publisher.
